# Identification of novel sublingual parameters to analyze and diagnose microvascular dysfunction in sepsis: the NOSTRADAMUS study

**DOI:** 10.1186/s13054-021-03520-w

**Published:** 2021-03-19

**Authors:** Alexandros Rovas, Jan Sackarnd, Jan Rossaint, Stefanie Kampmeier, Hermann Pavenstädt, Hans Vink, Philipp Kümpers

**Affiliations:** 1 Department of Medicine D, Division of General Internal and Emergency Medicine, Nephrology, and Rheumatology, University Hospital Münster, Albert-Schweitzer-Campus 1, 48149 Münster, Germany; 2 Department of Cardiology and Angiology, University Hospital Münster, Albert-Schweitzer-Campus 1, 48149 Münster, Germany; 3 Department of Anesthesiology, Intensive Care and Pain Medicine, University Hospital Münster, Albert-Schweitzer-Campus 1, 48149 Münster, Germany; 4 Institute of Hygiene, University Hospital Münster, Albert-Schweitzer-Campus 1, 48149 Münster, Germany; 5 Department of Physiology, Cardiovascular Research Institute Maastricht, Maastricht University, Maastricht, The Netherlands

**Keywords:** Endothelial glycocalyx, Capillary recruitment, Perfused boundary region, Microvascular health score, Sepsis

## Abstract

**Background:**

The availability of handheld, noninvasive sublingual video-microscopes allows for visualization of the microcirculation in critically ill patients. Recent studies demonstrate that reduced numbers of blood-perfused microvessels and increased penetration of erythrocytes into the endothelial glycocalyx are essential components of microvascular dysfunction. The aim of this study was to identify novel microvascular variables to determine the level of microvascular dysfunction in sepsis and its relationship with clinical variables.

**Methods:**

This observational, prospective, cross-sectional study included 51 participants, of which 34 critically ill sepsis patients were recruited from intensive care units of a university hospital. Seventeen healthy volunteers served as controls. All participants underwent sublingual videomicroscopy by sidestream darkfield imaging. A new developed version of the Glycocheck™ software was used to quantify vascular density, perfused boundary region (PBR-an inverse variable of endothelial glycocalyx dimensions), red blood cell (RBC) velocity, RBC content, and blood flow in sublingual microvessels with diameters between 4 and 25 µm.

**Results:**

A detailed analysis of adjacent diameter classes (1 µm each) of vessels between 4 and 25 µm revealed a severe reduction of vascular density in very small capillaries (5–7 µm), which correlated with markers of sepsis severity. Analysis of RBC velocity (V_RBC_) revealed a strong dependency between capillary and feed vessel V_RBC_ in sepsis patients (*R*^2^ = 0.63, *p* < 0.0001) but not in healthy controls (*R*^2^ = 0.04, *p* = 0.43), indicating impaired capillary (de-)recruitment in sepsis. This finding enabled the calculation of capillary recruitment and dynamic capillary blood volume (CBV_dynamic_). Moreover, adjustment of PBR to feed vessel V_RBC_ further improved discrimination between sepsis patients and controls by about 50%. By combining these dynamic microvascular and glycocalyx variables, we developed the microvascular health score (MVHS_dynamic_™), which decreased from 7.4 [4.6–8.7] in controls to 1.8 [1.4–2.7] in sepsis patients (*p* < 0.0001) and correlated with sepsis severity.

**Conclusion:**

We introduce new important diameter-specific quantification and differentiated analysis of RBC kinetics, a key to understand microvascular dysfunction in sepsis. MVHS_dynamic_, which has a broad bandwidth to detect microvascular (dys-) function, might serve as a valuable tool to detect microvascular impairment in critically ill patients.

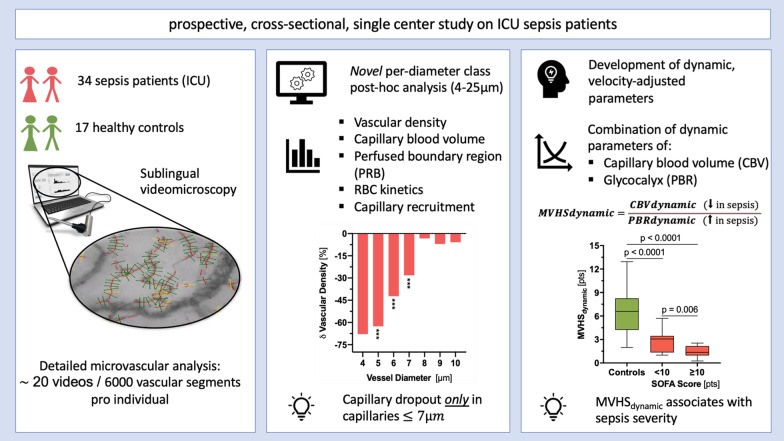

**Supplementary Information:**

The online version contains supplementary material available at 10.1186/s13054-021-03520-w.

## Background

Alterations of microvascular perfusion caused by endothelial cell dysfunction, glycocalyx degradation, increased leukocyte adhesion, microthrombus formation, and regional redistribution of blood flow contribute to organ failure in critically ill patients [1–3]. The use of handheld, noninvasive sublingual video microscopes, such as incident dark field (IDF) or sidestream dark field (SDF) microscopy, allows researchers to visualize and analyze microvascular perfusion alterations in vivo [4, 5]. Over the past decade, several measures of microvascular perfusion variables have been proposed to improve risk stratification, prognostication and eventually to individualize the therapy, especially in critically ill patients with sepsis and septic shock [6–13]. Despite tremendous efforts to improve video quality and standardize reporting, the quantitative analysis of functional measures remains challenging and is often performed manually [6]. Several visual scoring systems and specialized (semi-)automated analysis tools have been developed to shorten time-to-result and improve accuracy [14–17]. Common to all these approaches is the (automated) selection and pooled analysis of microvessels with a diameter < 20 (rarely < 10) µm. An improved spatiotemporal resolution within this < 20 µm range is desirable to further dissect and understand functional microvascular alterations in sepsis and critical illness.

We have previously used the Glycocheck™ System to analyze the perfused boundary region (PBR), an inverse variable of endothelial glycocalyx (eGC) dimensions, in sublingual microvessels from sepsis patients [18–20]. The software automatically detects, records, and analyzes the PBR according to diameter classes (each 1 µm) in microvessels with diameters between 4 and 25 μm. The methodology showed very good inter- and intra-observer reproducibility under real-life conditions [19] and excellent accuracy compared to other in vitro and ex vivo estimations of eGC thickness [18, 20, 21]. The aim of this study was to identify novel dynamic microvascular variables generated by a new diameter-class-wise approach to determine the level of microvascular dysfunction in sepsis and its relationship with established clinical variables and the number of dysfunctional organs.

## Materials and methods

### Study population

This prospective, observational, cross-sectional study took place in the medical and operative ICUs of the University Hospital Münster. The study was performed in accordance with the Declaration of Helsinki and was approved by the competent ethics committee (amendment to 2016-073-f-S). Some of the participants were already included in previous studies on eGC damage in sepsis [18, 20].

After written informed consent was obtained from the patients or their legal representatives, 34 adult ICU patients with sepsis were enrolled non-consecutively after initial resuscitation. Sepsis was defined by the sepsis-3 criteria published by the ESICM-SCCM Sepsis Redefinitions Task Force [22]. Exclusion criteria were age < 18 years, pregnancy, or oral mucosal inflammation or injury, which could locally influence the sublingual microvasculature. Seventeen apparently healthy volunteers served as controls.

Demographic variables, routine chemistry tests, and physiological variables, including the Sequential Organ Failure Assessment (SOFA) score [22] and a contemporary version of the Charlson Comorbidity Index (CCI) [23], were obtained for each subject at the time of sublingual videomicroscopy (Table 1). Each videomicroscopy set consisted of two complete measurements (see below) which were averaged to account for spatial heterogeneity of the sublingual microvasculature.

**Table 1:** Baseline characteristics

Variable	Healthy controls	Sepsis patients	*p* value
Number of participants (*n*; %)	17	34	–
Female sex (*n*; %)	9 (53)	8 (24)	0.06
Age (years, median (IQR))	51 (33–72)	66 (56–78)	0.06
BMI (kg/m^2^, median (IQR))	23 (21.5–24.9)	25.8 (21.9–29.2)	0.06
Duration of sepsis at study inclusion (days, median (IQR))*	–	2 (1–4)	–
SOFA score (median (IQR))	–	10 (8–13)	–
Number of dysfunctional organs (median (IQR))	–	4 (3–5)	–
Organ replacement therapy (*n*; %)	–	22 (64.7)	–
Mechanical ventilation (*n*; %)	–	21 (61.8)	–
Acute dialysis (*n*; %)	–	6 (17.7)	–
Vasopressors (*n*; %)	–	26 (76.4)	–
Norepinephrine dose (μg/kg/min)	–	0.06 (0.02–0.14)	–
Septic shock (*n*; %)*	–	6 (17.7)	–
Inhospital mortality (*n*; %)		13 (38.2)	–
CCI score (median (IQR))	–	2 (1–3)	–
*Macrocirculation data* (median (IQR))			
MAP (mmHg)	95.3 (86–98.7)	71.8 (66.5–81.1)	< 0.0001
Heart Rate (pulse/min)	72 (65–83)	90 (75–102)	0.0002
Respiratory Rate (breaths/min)	14 (12–15)	21 (18–26)	< 0.0001
Temperature (°C)	36.6 (36.5–36.9)	36.9 (36.4–37.7)	0.08
*Laboratory data* (median (IQR))			
CRP (mg/dl)	0.5	22.4 (16.9–33.3)	< 0.0001
IL-6 (ng/ml)	2 (2–3)	452 (137–1121)	< 0.0001
PCT (ng/ml)	0.05 (0.04–0.07)	17.43 (2.04–52.44)	< 0.0001
pH	–	7.41 (7.35–7.45)	–
Lactate (mmol/l)	1.1 (0.80–1.4)	1.70 (1.08–2)	0.004

*BMI* Body mass index, *CCI score* Charlson Comorbidity Index, *CRP* C-reactive protein, *IL-6* Interleukin-6, *IQR* interquartile range, *MAP* Mean arterial pressure, *PCT* Procalcitonin, *SOFA* score Sequential Organ Failure Assessment score

*Septic shock: Vasopressors required to maintain MAP ≥ 65 mmHg AND serum lactate > 2 mmol/l. *p* value was calculated between healthy controls and sepsis patients

### Video acquisition and analysis of the sublingual microvasculature

**Fig. 1:**
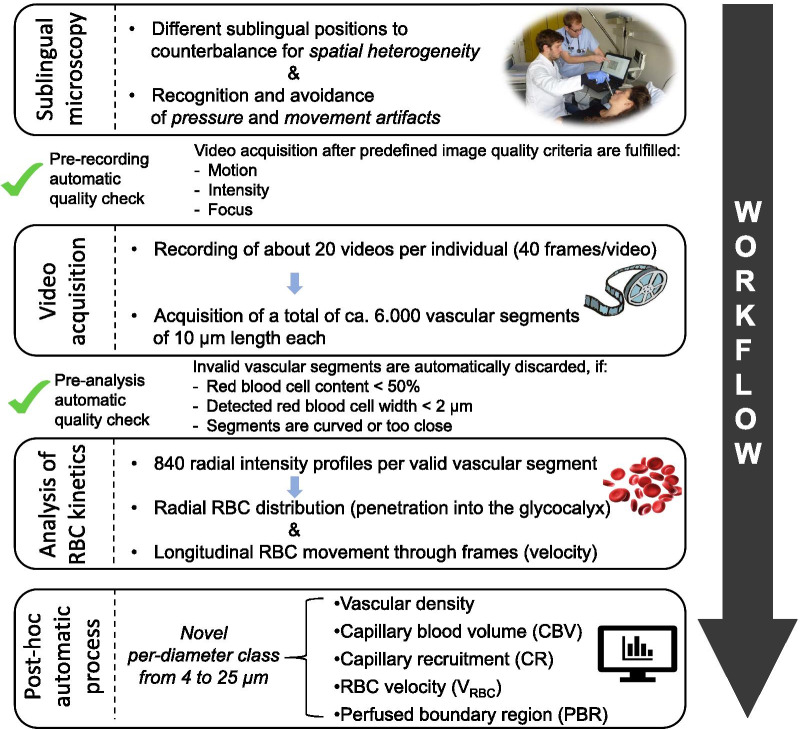
Flowchart showing the process of video acquisition and data analysis. *D* = Vessel Diameter, in µm

Figure 1 provides an overview of the process of video acquisition, data analysis and post-processing. Bedside intravital microscopy was performed with a sidestream dark field (SDF) camera (CapiScope HVCS, KK Technology, Honiton, UK) to visualize the sublingual microvasculature according to a standardized procedure as described in detail previously [20]. A physician experienced in the SDF technique, trained to recognize and avoid pressure artifacts performed the image acquisition based on the current round table recommendations [6]. The SDF camera uses green light-emitting stroboscopic diodes (540 nm) to detect the hemoglobin of passing red blood cells (RBCs). Using a 5 × objective with a 0.2 numerical aperture, images were captured, providing a 325-fold magnification in 720 × 576 pixels at 23 frames per second as described in detail previously [19, 24, 25].

The analysis of all variables was carried out exclusively with the GlycoCheck™ Software (Microvascular Health Solutions Inc., Salt Lake City, UT, USA) software. Briefly, the GlycoCheck™ Software allows video acquisition after predefined image quality criteria (motion, intensity, and focus) are fulfilled. Specifically, light intensity range is automatically set to prevent under- or over-exposure of the video. The acceptable motion range is set so that every vessel can be tracked in all frames of each video. Each complete measurement consists of at least ten 2-s videos (40 frames/video), containing a total of about 3000 vascular segments of 10 μm length each. All videos are deliberately obtained from different positions to counterbalance spatial heterogeneity of the sublingual microcirculation. The software automatically subjects the obtained vascular segments to a strict quality check, as previously described in detail [25]. Briefly, vascular segments are considered valid only if: (1) red blood cell content is ≥ 50%, (2) RBC width is ≥ 2 µm, (3) segments are neither curved, (4) nor too close to each other. Invalid vascular segments are marked yellow and are automatically discarded, while all valid vascular segments (green lines) are further analyzed. Therefore, all further parameters are calculated based on these valid segments. Screenshots of the sublingual mucosa of five randomly chosen individuals are shown in Additional file 1: Fig. S1 (with and without automatically acquired vessel detection and quality check). Moreover, two additional movie files illustrate the sublingual mucosa of a healthy control and a sepsis individual with and without automatic vessel detection and subsequent quality check in more detail (Additional file 2: Video 1, Additional file 3: Video 2). Finally, the software obtains up to 840 radial intensity profiles for each valid vascular segment based on the RBC column width (RBCW), and automatically groups vessels from 4 to 25 µm diameter in 22 separate diameter classes (1 µm each). This concept has been successfully used and validated in the past [25]. Data from two complete measurements (hereafter referred to as “measurement set”) were extracted, analyzed, and averaged offline to avoid sampling error and to counterbalance spatiotemporal heterogeneity of the sublingual microcirculation [19, 20].

#### RBC velocity

Valid red blood cell (RBC) velocities (V_RBC_) are determined in individual vessel segments in an automatic fashion by cross correlation of longitudinal RBC intensity profiles between frames of recorded videos. A correlation coefficient of ≥ 0.80 was required to ensure accurate estimates of longitudinal RBC displacement. RBC velocity is determined by dividing RBC displacement by the time between video frames and expressed as µm/s. The automatic estimation of the V_RBC_ correlated excellently with a manual analysis (*R*^2^ = 0.94, *p* < 0.0001; Additional file 4: Fig. S2).

#### Perfused vascular and capillary density

An absolute measure for valid perfused vascular density (mm/mm^2^) can be determined from the number of vascular segments containing RBCs multiplied by capillary segment length (each 10 µm). All detected RBC-containing vessel segments (RBC content ≥ 50%) with V_RBC_ ≥ 0 µm/s were automatically counted in the video recordings of each subject. Vascular density was normalized to tissue surface area. As *non-perfused* vessels (i.e., without RBCs present), as well as vessels not meeting the quality criteria cannot be detected by our methodology, therefore vascular density in this manuscript refers to *valid perfused* vascular density (hereafter vascular density). *Capillary* density was defined as vascular density of vessels with a diameter equal or smaller than the diameter of a single red blood cell (RBC diameter ~ 7 to 8 µm [26]; capillary density *D* ≤ 7 µm).

#### Absolute and static capillary blood volume

An absolute measure for valid capillary blood volume (CBV_absolute_) can be determined from the number of capillary segments multiplied by capillary segment length (i.e., capillary density (mm/mm^2^)) and segment-specific capillary cross-sectional area ($\pi$ * radius^2^). In addition to counting the number of RBC-containing capillary segments, a functional estimate of CBV relative to larger vessel blood volume can be determined by measuring average V_RBC_ in capillaries and larger blood vessels (hereafter feed vessels). The corresponding V_RBC_ ratio in feed vessels (diameter 10 to 25 µm, hereafter *D* ≥ 10 µm) over V_RBC_ in capillaries (diameter 4 to 7 µm, hereafter *D* ≤ 7 µm) denotes the CBV ratio ($= V_{RBC} \left( {D \ge 10\,\upmu {\text{m}}} \right)/V_{RBC} \left( {D \le 7\,\upmu {\text{m}}} \right)$). In short, an increase in capillary blood volume relative to feed vessel blood volume will *reduce* capillary V_RBC_ and this will *increase* the CBV ratio. Multiplying the CBV_absolute_ with the CBV ratio gives the static capillary blood volume (CBV_static_), which is defined as: $CBV_{static} = CBV_{absolute} *V_{RBC} \left( {D \ge 10\,\upmu {\text{m}}} \right)/V_{RBC} \left( {D \le 7\,\upmu {\text{m}}} \right)$.

#### Capillary recruitment and dynamic capillary blood volume

To take the ability to recruit additional capillaries into account, an estimate can be made of the capillary recruitment (CR) by measuring the slope of the relationship between V_RBC_ (*D* ≤ 7 µm) and V_RBC_ (*D* ≥ 10 µm). When the number of blood perfused capillaries increases upon and increase in V_RBC_ (*D* ≥ 10 µm), the accompanying increase in V_RBC_ (*D* ≤ 7 µm) will be less than proportional (i.e., the regression slope will be < 1) and capillary recruitment can be defined as 1 − slope(V_RBC_ (*D* ≤ 7 µm), V_RBC_ (*D* ≥ 10 µm)). Two extreme examples of this concept are shown in Additional file 5: Fig. S3: In case capillary blood volume doubles when large vessel RBC velocity increases twofold, the slope(V_RBC_ (*D* ≤ 7 µm), V_RBC_ (*D* ≥ 10 µm)) will be 0 and CR = 1 − slope 0 = 1 = 100%. In the absence of changes in capillary blood volume when V_RBC_ (*D* ≥ 10 µm) increases twofold, capillary RBC velocities are expected to also change proportionally by twofold, and the slope of V_RBC_ (*D* ≤ 7 µm) vs. V_RBC_ (*D* ≥ 10 µm) will be 1 and CR = 1 − slope 1 = 0 = 0%. Multiplying the CBV_static_ * (1 + CR) gives the dynamic capillary blood volume (CBV_dynamic_).

#### Static and dynamic perfused boundary region (PBR)

The software calculates the dynamic lateral movement of RBCs into the permeable part of the eGC layer, expressed as the PBR (in μm). An impaired eGC permits a greater number of RBCs to penetrate deep into the endothelium, which is translated as an increase in the PBR value. The radial distribution of RBCs in each valid segment defines the median RBC width (RBCW), as well as the outer edge of the RBC-perfused vessel diameter (D_perf_). The PBR_static_ is defined as the distance between the RBCW and D_perf_ and is calculated using the following formula: $\left( {Dperf {-} RBCW} \right)/2$ [25].

In line with the above-described measures of velocity-dependent increases in capillary blood volume, it is possible that also penetration of RBCs into the luminal glycocalyx surface (as reflected by PBR) is velocity-dependent. To minimize possible flow-dependent variability in PBR estimation, the slope(PBR_static_, V_RBC_ (*D* ≥ 10 µm)) can be used to PBR_dynamic_ under equal conditions, i.e., the absence of RBC velocity (V_RBC_ (*D* ≥ 10 µm) set to 0 µm/s) (Additional file 6: Fig. S4 for details).

### Statistical analysis

Data are presented as absolute numbers, percentages, or medians with corresponding 25th and 75th percentiles (interquartile range; IQR), as appropriate. The nonparametric Mann–Whitney U test and the chi-square test were used to compare variables between patients and controls. To correct for multiple testing in comparisons of microcirculation variables per-diameter class we used the false discovery rate (FDR) approach of Benjamini, Krieger and Yekutieli, setting a *q*value < 0.05 as significant. Spearman rank correlation coefficient was used to assess correlations between clinical and microvascular variables. Associations between microvascular measures were evaluated using simple and adjusted linear regression models. All the tests used were two-sided, and statistical significance was set at *p* < 0.05. Our study was powered to detect a moderate correlation (Spearman correlation coefficient = 0.5) between microvascular health score and SOFA score in the septic cohort with 85% power given a two-sided alpha of 0.05 [27]. SPSS version 26 (IBM Corporation, Armonk, NY, USA) and GraphPad Prism version 8.4.3 (GraphPad Prism Software Inc., San Diego, CA, USA) were used for statistical analyses and preparation of figures.

## Results

The clinical and demographic characteristics of the 51 study participants are shown in Table 1 and Additional file 7: Table A1. 50% of the patients had a respiratory focus of infection, while the remainder demonstrated other etiologies (Additional file 7: Table A1). From a total of 34 sepsis patients, 22 (65%) required organ replacement therapy (mechanical ventilation or/and renal replacement therapy) and 26 (76%) were vasopressor-dependent at study inclusion. Our sepsis cohort had a median (IQR) SOFA score of 10 (8–13), indicating moderate disease severity. The median time of sepsis begin was 2 days (1–4) and 6 (18%) patients had septic shock (Sepsis-3 definitions: lactate > 2 mmol/l and need for vasopressors to maintain a mean arterial pressure ≥ 65 mm Hg) at the time of study inclusion.

### Analysis of sublingual microcirculation according to vessel diameter class

**Fig. 2:**
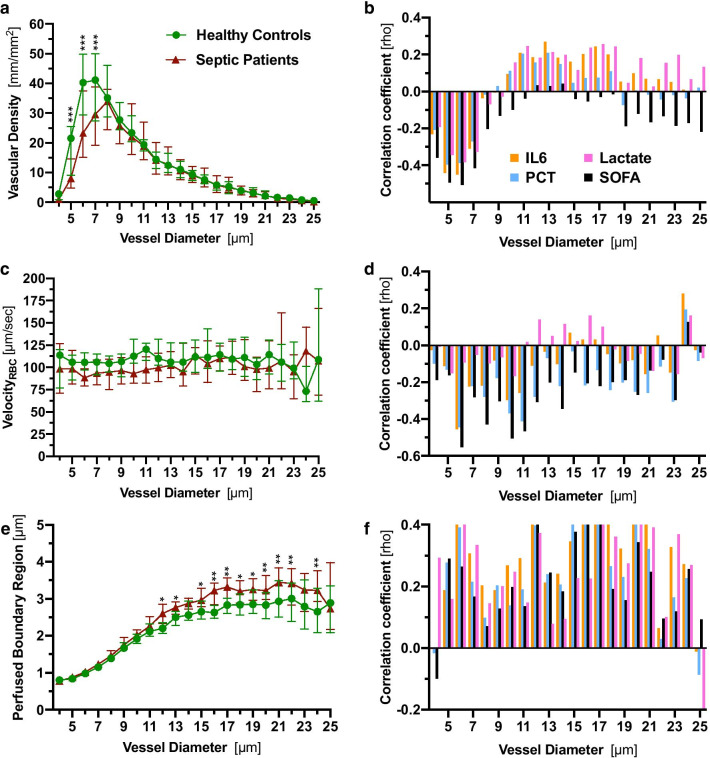
Analysis of sublingual microcirculation according to vessel diameter class. **a**, **c**, **e** Median and IQR values of vascular density, PBR_static_ values and RBC velocity of healthy controls and sepsis patients according to diameter class from 4 to 25 µm. **b**, **d**, **f** Bar charts showing the correlation coefficient (Spearman) between microvascular and clinical variables. *IQR* inter quartile range, *IL6* interleukin 6, *PBR* perfused boundary region, *PCT* procalcitonin, *RBC* red blood cell, *SOFA score* sequential organ failure assessment score. *Q* value (adjusted *p* value): *q < 0.05, **q < 0.01, ***q < 0.001

First, we compared vascular density, V_RBC_ and PBR_static_ between healthy controls and sepsis patients in a diameter-class-wise fashion. This approach revealed a statistically significant decrease in vascular density only in the diameter classes 5, 6 and 7 µm in sepsis patients (− 63%, − 42%, and − 28% compared to controls), whereas the remaining diameter classes from 8 to 25 µm were not different between the groups (Fig. 2a and Additional file 8: Fig. S5). However, vascular density was not different between the groups in a pooled analysis (4–25 µm range) (12.3 vs. 12.8 mm/mm^2^, *p* = 0.34). To check our results for plausibility, we correlated, in an explorative manner, vascular density with clinical variables, such as interleukin-6 (IL-6), procalcitonin (PCT), lactate and SOFA score. We found a robust inverse correlation in the 4 to 7 µm diameter range, while these associations were absent or even partially reversed in larger vessels (Fig. 2b and Additional file 7: Table A2).

V_RBC_ showed a huge overlap between patients and controls. Median V_RBC_ trended to be slightly lower in sepsis patients, especially in smaller vessels and was inversely correlated with variables of disease severity (Fig. 2c, d and Additional file 7: Table A2). A significant sepsis-induced increase in PBR_static_ was particularly apparent in feed vessels, probably due to loss of the affected smaller vessels (lower capillary density) (Fig. 2e and Additional file 9: Fig. S6). PBR_static_ values of individual diameter classes showed weak-to-moderate positive correlations with variables of sepsis severity (Fig. 2f and Additional file 7: Table A2).

Taken together, the diameter class-wise data analysis revealed individual sepsis-induced perturbations in vascular density, V_RBC_ and PBR_static_.

### Derivation of capillary recruitment and dynamic capillary blood volume.

**Fig. 3:**
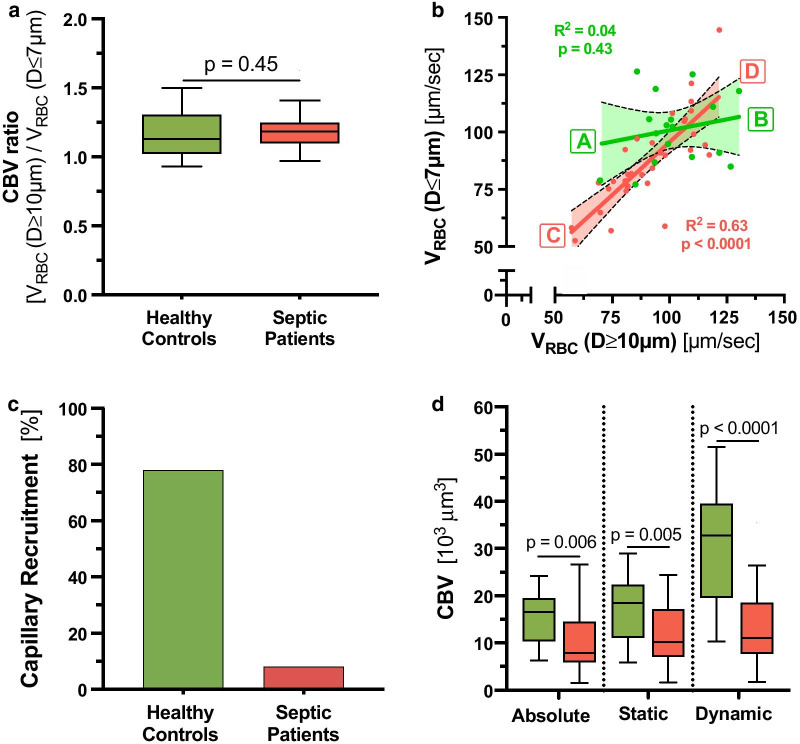
Derivation of capillary recruitment and dynamic capillary blood volume. **a** Box plots showing capillary blood volume (CBV) ratio in healthy controls (green) and sepsis patients (red). CBV ratio denotes the RBC velocity (V_RBC_) in feed vessels (*D* ≥ 10 µm) over V_RBC_ in capillaries (*D* ≤ 7 µm). **b** Scatter dot plots and simple linear regression (slope) with 95% confidence intervals of V_RBC_ in capillaries (*D* ≤ 7 µm) plotted against V_RBC_ in feed vessels (*D* ≥ 10 µm). Different states at the ends of the slope lines (indicated by green/red bold letters A-D) are further explained in Fig. 5. **c** Bar charts showing the capillary recruitment (CR), defined as 1 − slope (V_RBC_ (*D* ≤ 7 µm) vs. V_RBC_ (*D* ≥ 10 µm)) per group. **d** Box plots showing the development of different measures and estimates of CBV. *Left:* CBV_absolute_ is determined from the number of capillary segments multiplied by capillary segment length (i.e., capillary density (mm/mm^2^)) and segment-specific capillary cross-sectional area (π * radius^2^). Briefly, as each vascular segment can be considered a cylinder, the segment-specific capillary cross-sectional area can be calculated with the mathematical type π * radius^2^ (circle’s area). The radius is estimated every 10 µm (segment’s length) and recorded accordingly. *Middle:* CBV_static_ is calculated as CBV_absolute_ * V_RBC_ (*D* ≥ 10 µm)/V_RBC_ (*D* ≤ 7 µm). *Right:* CBV_dynamic_ is defined as CBV_static_ * (1 + CR). *RBC* red blood cell, *CBV* capillary blood volume, *CR* capillary recruitment, *D* diameter, *V* velocity

Having observed a significant decrease in vascular density in very small capillaries (diameter (*D*) ≤ 7 µm) in sepsis patients, we determined and focused on the capillary blood volume (Fig. 3). CBV_absolute_ decreased from 16.5 [10.3–19.4] 10^3^μm^3^ in healthy controls to 7.9 [5.9–14.5] 10^3^μm^3^ in sepsis patients (*p* = 0.006). To further improve discrimination between the groups, we added some functional variables to the CBV calculation.

First, we calculated CBV_static_, by multiplying CBV_absolute_ with a ratio derived from V_RBC_ in feed vessels relative to capillaries (V_RBC_ (*D* ≥ 10 µm)/V_RBC_ (*D* ≤ 7 µm)). However, the suspected decrease in the CBV ratio (due to decrease in capillary density) in sepsis patients did not occur (Fig. 3a). The reason for this only became apparent after plotting capillary V_RBC_ (*D* ≤ 7 µm) as a function of large vessel V_RBC_ (*D* ≥ 10 µm) (Fig. 3b). This type of analysis revealed a strong dependency between V_RBC_ (*D* ≤ 7 µm) and V_RBC_ (*D* ≥ 10 µm) in sepsis patients (*R*^2^ = 0.63, *p* < 0.0001), indicating impaired capillary (de-)recruitment in this group. In contrast, capillary V_RBC_ is relatively constant in healthy controls as reported before [28], indicating functioning (de-)recruitment of CBV associated with changes of feed vessel blood flow in healthy subjects. CBV_static_ decreased from 18.5 [11.1–22.3] 10^3^μm^3^ in healthy controls to 10.2 [7.1–17.1] 10^3^μm^3^ in sepsis patients (*p* = 0.005).

Next, we calculated capillary recruitment (CR) as 1- the slope(V_RBC_ (*D* ≤ 7 µm), V_RBC_ (*D* ≥ 10 µm)). CR per group decreased from 78% in healthy controls to 8% in sepsis patients (Fig. 3c). Finally, we calculated CBV_dynamic_ by multiplying CBV_static_ with (1 + CR). CBV_dynamic_ showed a significant higher and much wider *normal* range in healthy controls compared to CBV_absolute_ and CBV_static_, respectively. CBV_dynamic_ decreased from 32.8 [19.7–39.5] 10^3^μm^3^ in healthy controls to 11.1 [7.7–18.5] 10^3^μm^3^ in sepsis patients (*p* < 0.0001). Overall, we were able to show that by adding dynamic variables to CBV calculation, the discrimination between the groups could be significantly improved (Fig. 3d).

### Derivation of an RBC velocity-adjusted perfused boundary region

**Fig. 4:**
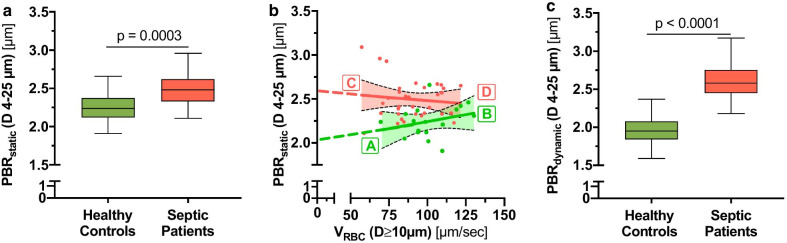
Derivation of an RBC velocity-adjusted perfused boundary region. **a** Box plots showing PBR_static_ (D 4 to 25 µm) in healthy controls (green) and sepsis patients (red). **b** Scatter dot plots and simple linear regression (slope) with 95% confidence intervals of PBR_static_ (D 4 to 25 µm) plotted against V_RBC_ in feed vessels (*D* ≥ 10 µm). Different states at the ends of the slope lines (indicated by green/red bold letters A-D) are further explained in Fig. 6. **c** Box plots of PBR_dynamic_, a velocity-adjusted estimate of the PBR where the V_RBC_ (*D* ≥ 10 µm) was set to 0 µm/s. *RBC* red blood cell, *CBV* capillary blood volume, *D* diameter, *V* velocity

We have previously shown that PBR values (averaged across all diameter classes from 5 to 25 μm) increase in sepsis patients [18–20], indicating sepsis-induced damage of the eGC. In this study, PBR_static_ (D 4 to 25 µm) increased from 2.24 [2.13–2.35] µm in healthy controls to 2.48 [2.33–2.62] µm in sepsis patients (*p* < 0.0001) (Fig. 4a). In sepsis patients, PBR_static_ showed a tendency to *in*crease at low levels of feed vessel velocity, whereas in healthy controls, PBR_static_ showed a tendency to stay constant or even slightly *de*crease at low feed vessel velocity (Fig. 4b). To eliminate the influence of different RBC velocities on PBR_static_, we estimated PBR_dynamic_ at a feed vessels V_RBC_ of 0 µm/s based on the different slopes(PBR_static_, V_RBC_ (*D* ≥ 10 µm)) in healthy subjects and sepsis patients (Fig. 4b and Additional file 6: Fig. S4). PBR_dynamic_ increased from 1.95 [1.84–2.08] µm in healthy controls to 2.58 [2.43–2.76] µm in sepsis patients (*p* < 0.0001) (Fig. 4c). In summary, adjustment of the PBR to V_RBC_ improved discrimination between the groups by about 50%.

### Development of the Microvascular Health Score

**Fig. 5:**
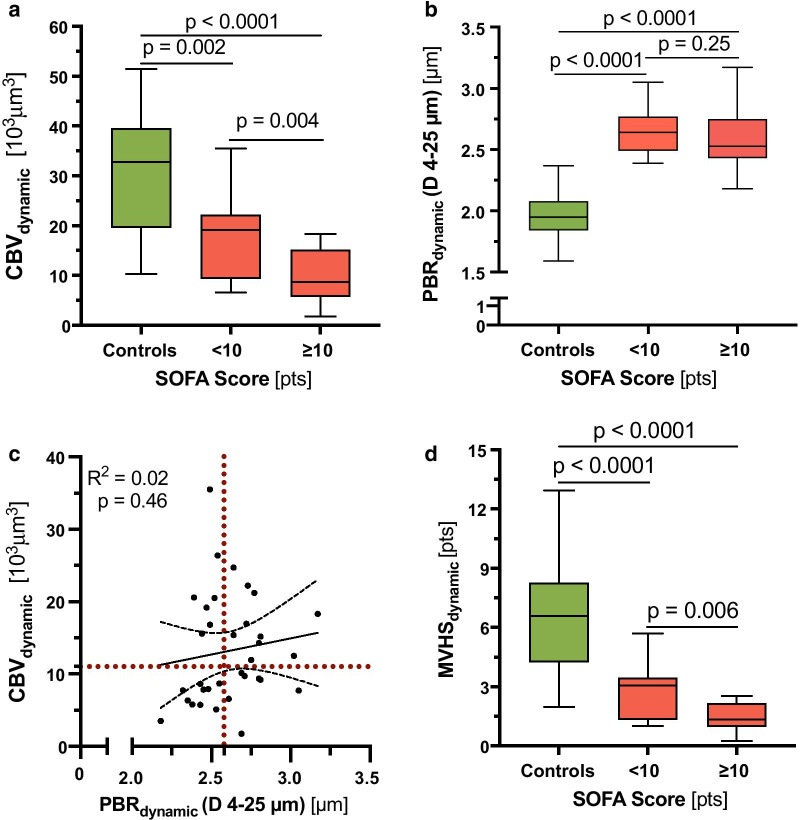
Association of the CBV_dynamic_, PBR_dynamic_ and dynamic Microvascular Health Score (MVHS_dynamic_) with disease severity. Association of **a** CBV_dynamic_ and **b** PBR_dynamic_ with sequential organ failure assessment score (SOFA) score after dichotomizing (median) the sepsis group. **c** Scatter dot plots and simple linear regression (slope) with 95% confidence intervals of CBV_dynamic_ plotted against PBR_dynamic_ (D 4 to 25 µm) in the septic population. The red dotted lines represent the median values of CBV and PBR, respectively. **d** Association of MVHS_dynamic_ with sequential organ failure assessment score (SOFA) score after dichotomizing (median) the group

We have previously shown, that glycocalyx damage and microcirculatory impairment do neither coincide, nor do they occur in proportion in every sepsis patient [20]. This un-coupling of changes in PBR and CBV can also be reproduced in the group of sepsis patients (Fig. 5a–c). To account for this finding, we combined the different variables into one Microvascular Health Score (MVHS™) (Additional file 10: Fig. S7). Because CBV *de*creases and PBR increases in sepsis, we formed the quotient of CBV/PBR to calculate the MVHS in a static and a dynamic version (MVHS_static_ = CBV_static_ (D 4 to 6 μm)/PBR_static_ (D 4 to 25 μm) and MVHS_dynamic_ = CBV_dynamic_ (D 4 to 6 μm)/PBR_dynamic_ (D 4 to 25 μm)).

The median MVHS_static_ was significantly lower in sepsis patients 1.70 [1.32–2.53] points compared to healthy controls 4.03 [2.11–4.33] points (*p* < 0.001). The median MVHS_dynamic_ stressed the difference between sepsis patients and healthy controls (1.78 [1.38–2.67] vs. 7.43 [4.65–8.73] points, *p* < 0.0001) even more than the MVHS_static_.

### Association of the Microvascular Health Score (MVHS) with disease severity

Both, MVHS_static_ and MVHS_dynamic_ correlated moderate-to-strong with SOFA score, number of dysfunctional organs, lactate, CRP, IL-6 and PCT (all *p* < 0.001) in a pooled analysis that included healthy controls and sepsis patients (Table 2). These observations remained unchanged in additional sensitivity analyses (Additional file 7: Table A3).

**Table 2:** Correlations of MVHS with clinical variables

Variable	Controls & sepsis patients (*n* = 51)		Sepsis patients (*n* = 34)	
	MVHS_static_	MVHS_dynamic_	MVHS_static_	MVHS_dynamic_
SOFA Score (points)	− 0.57 (*p* < 0.0001)	− 0.77 (*p* < 0.0001)	− 0.43 (*p* = 0.01)	− 0.44 (*p* = 0.01)
Number of dysfunctional organs (*n*)	− 0.60 (*p* < 0.0001)	− 0.80 (*p* < 0.0001)	− 0.51 (*p* = 0.002)	− 0.52 (*p* = 0.002)
Lactate (mmol/l)	− 0.37 (*p* = 0.007)	− 0.42 (*p* = 0.002)	− 0.23 (*p* = 0.19)	− 0.22 (*p* = 0.20)
CRP (mg/dl)	− 0.39 (*p* = 0.005)	− 0.61 (*p* < 0.0001)	0.02 (*p* = 0.92)	0.02 (*p* = 0.92)
IL6 (ng/ml)	− 0.48 (*p* = 0.001)	− 0.69 (*p* < 0.0001)	− 0.14 (*p* = 0.48)	− 0.14 (*p* = 0.48)
PCT (ng/ml)	− 0.47 (*p* < 0.0001)	− 0.68 (*p* < 0.0001)	− 0.27 (*p* = 0.13)	− 0.27 (*p* = 0.13)

Spearman correlation was used. The *p* values are indicated in brackets

*CRP* C-reactive protein, *IL-6* interleukin-6, *MVHS* microvascular health score, *PCT* procalcitonin, *SOFA Score* sequential organ failure assessment score

In the subgroup of sepsis patients, the correlations of MVHS_static_ and MVHS_dynamic_ with SOFA score and number of dysfunctional organs remained significant (all *p* ≤ 0.01) (Table 2 and Additional file 7: Table A4). MVHS_dynamic_ was significantly different in sepsis subgroups stratified by either median SOFA score or median number of dysfunctional organs, respectively (Fig. 5d and Additional file 11: Fig. S8).

## Discussion

In this study, we employed a differentiated, diameter class-wise analysis of RBC kinetics to identify novel variables of microvascular dysfunction in sepsis. This approach revealed several size- and group-specific characteristics of the measured variables within the diameter-range from 4 to 25 µm. Accordingly, we propose the MVHS_dynamic_, which has a broad bandwidth to detect microvascular (dys-)function in healthy subjects and critically ill patients. To our knowledge, the < 20 µm range has neither been divided into single µm-steps nor individually examined and compared with clinical information from healthy and septic subjects before.

### Capillary recruitment

**Fig. 6:**
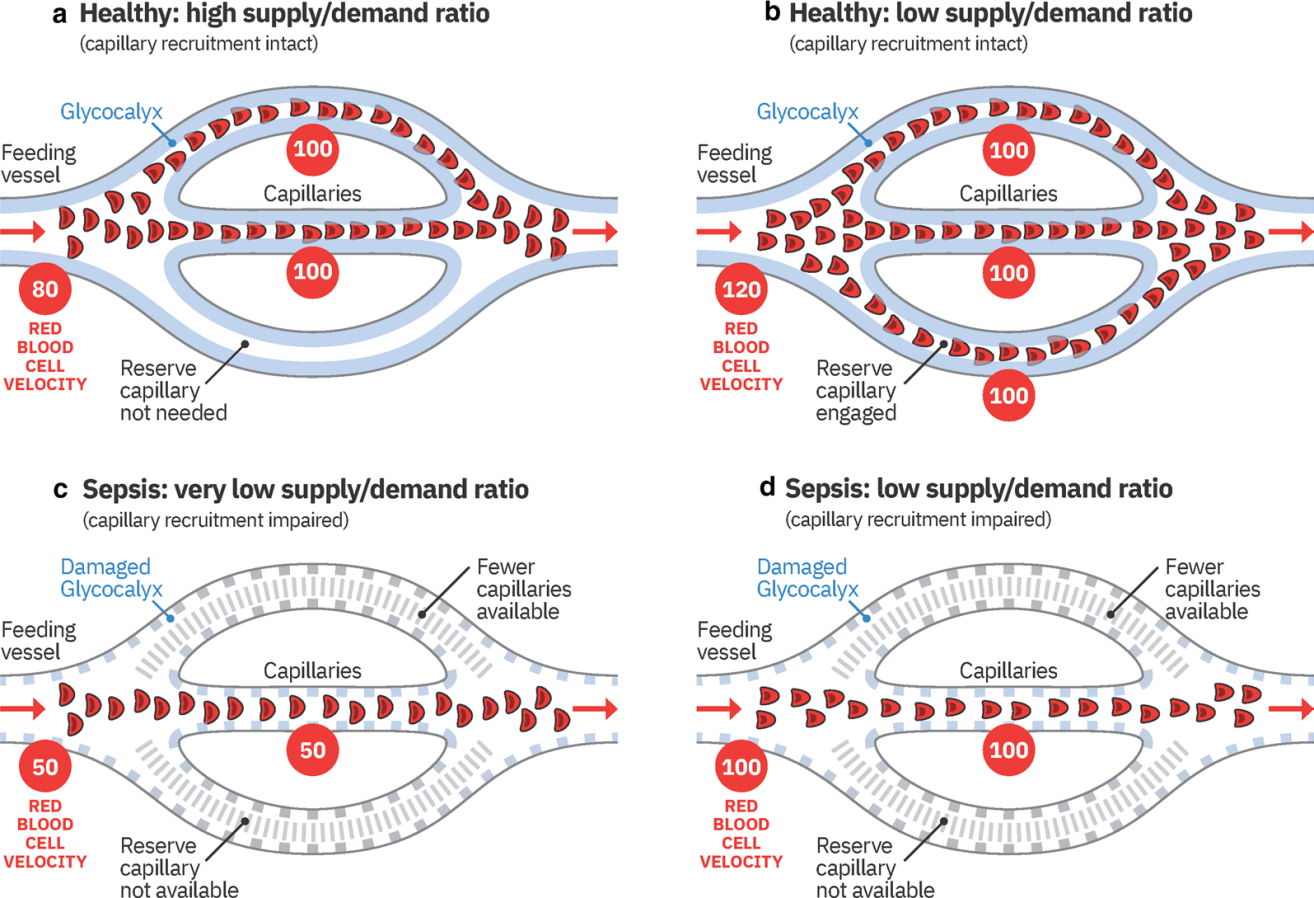
Cartoon showing the pathophysiologic concept of capillary (de-)recruitment in healthy conditions (**a**, **b**) and during sepsis (**c**, **d**). The respective ends of the spectrum of actual measured RBC velocities are indicated in the red circles. Conditions shown in A-D refer to corresponding green/red bold letters in Fig. 3B). In theory, higher RBC velocity states (low supply demand ratio, **b**, **d**) should go along with slightly thicker glycocalyx than lower RBC velocity states (high supply demand ration **a**, **b**). Furthermore, RBC flux is actually controlled by arterioles rather than precapillary sphincters. These details have been omitted for the sake of clarity

Observing that RBC dynamics differ between capillaries (*D* ≤ 7 µm) and feeding vessels (*D* ≥ 10 µm) led us to relate capillaries and feeding vessels to each other—which resulted in the calculation of capillary recruitment. The concept of capillary recruitment in skeletal muscle was first proposed by August Krogh in 1919 [29]. Krogh hypothesized that the opening of previously closed muscle capillaries, i.e., capillary recruitment, would allow capillary RBC velocities to remain low, and capillary oxygen extraction thereby efficient, despite large increases in blood supply. While the binary distinction between "closed" and "open" capillaries, was key to Krogh's model argument, modern observations suggest that capillary recruitment should be regarded rather in the context of continuous than binary changes in RBC distributions and velocities among capillaries [30, 31]. In this respect, our quantitative data show that healthy individuals (at least under resting conditions) have relatively constant median capillary RBC velocities, which seems to be independent of the associated V_RBC_ in the feed vessels. Even though the underlying (auto-) regulation mechanisms remain incompletely understood, compliance with a narrow RBC velocity range seems plausible to guarantee an optimal supply–demand ratio in capillaries. Conceptually, *constant* capillary RBC velocities in the face of varying V_RBC_ in larger feeding vessels is consistent with a) increasing numbers of RBC-perfused capillaries at tissue sites with higher metabolic demand and increased blood supply in feeding vessels, as well as b) reduced numbers of RBC-perfused capillaries at sites with lower metabolic demand and reduced feeding vessel blood supply. The failure to maintain constant RBC velocities in capillaries of patients with sepsis and the fact that capillary RBC velocities in our septic cohort change proportionally with RBC velocity changes in feedings vessels, reflects that the number of perfused capillaries in sepsis patients is *fixed* and insensitive to local variations in tissue metabolic demand. A simplified version of the pathophysiologic concept of capillary (de-)recruitment observed in this study is visualized in Fig. 6.

Estimates of capillary recruitment in our cohort are based on *per group* analysis of relations between RBC velocities in capillaries and feeding vessels. New recording and analysis strategies that allow for calculation of capillary recruitment on a per-patient basis are currently under investigation. This modification will allow for capturing enough low- and high flow situations per subject to generate intra-individual regression slopes. Thus, it will be possible to determine the capillary recruitment *per individual subject*, which might further improve discrimination of dynamic variables, such as CBV_dynamic_ and MVHS_dynamic_.

### Endothelial glycocalyx damage

The sepsis-associated PBR increase measured in this study is consistent with changes observed in previous studies [18–20, 32–34]. The current study shows that adjustment of PBR estimates to V_RBC_ improved discrimination between the groups by about 50%. This gain in discriminatory power is important, as PBR estimates have so far shown a considerable overlap between healthy controls and sepsis patients. This adjustment accounts for RBC velocity dependency of the glycocalyx [35, 36]. Especially in sepsis patients, PBR tends to increase when RBC velocity decreases, suggesting that glycocalyx extends towards a more porous and permeable compartment in the absence of exposure to fluid/RBC shear forces. The fact that PBR increases are more extreme in the sepsis patients, is consistent with previous studies showing that glycocalyx is damaged and more permeable in sepsis, which likely contributes to its increased RBC velocity dependency. However, it is difficult to estimate if the higher PBR observed in sepsis patients is caused solely by real glycocalyx damage (i.e., enzymatic shedding [18, 37]) or if temporary and functional changes that enable nutrient and solute supply to the interstitial space contribute to that PBR increase. We have previously shown that PBR estimates correlate excellent with glycocalyx thickness measured by atomic-force microscopy in cultured endothelial cells exposed to concordant sera from healthy subjects and sepsis patients [18, 20]. Glycocalyx damage in vitro was completely abolished when enzymatic activity of heparanase, a heparan sulphate specific endo-beta-D-glucuronidase, was blocked by addition of heparin [18]. Furthermore, circulating levels of Syndecan-1, a core protein of the intact glycocalyx, increased tenfold in sepsis patients and correlated with PBR values [20], indicating that enzymatic shedding is a dominant mechanism of PBR increase in sepsis. Elegant work in the cremasteric microcirculation showed that, inflammation-induced glycocalyx shedding increases effective capillary diameter. This effect is, however, defeated by enhanced white blood cell (WBC)-endothelial interactions and subsequent venular WBC obstruction, resulting in a *net* reduction of capillary V_RBC_ [38]. Glycocalyx damage may therefore partly explain the trend towards lower V_RBC_ in capillaries.

### Microvascular health score

In the current cross-sectional cohort, the proposed MVHS_static_ and MVHS_dynamic_ did correlate with SOFA score and number of dysfunctional organs in the subgroup of sepsis patients. Correlations between classical variables of the microcirculation and the SOFA score have been rarely reported. The SOFA correlated negatively with microvascular flow indices (MFI) as well as total and perfused vascular densities (TVD and PVD) in sepsis patients receiving activated protein C [33]. However, we couldn´t detect any significant correlations of MFI, TVD or PVD with the SOFA score in a previous sepsis study [20]. The finding that the MVHS performed better than its individual components is a strong argument for combining different measures of microvascular dysfunction such as vascular blood volume (CBV) and glycocalyx properties (PBR) into one score. In our view, in particular the combination of CBV and PBR makes sense, because CBV and PBR appear to behave independently of each other [20], and may thus indicate different aspects of microvascular dysfunction. To clarify whether the MVHS can indeed predict relevant outcome in sepsis patients, we initiated two prospective, observational, longitudinal studies to evaluate the MVHS in the emergency room (Early Detection of Glycocalyx Damage in Emergency Room Patients—the EDGE Study, Clinicaltrials.gov Identifier: NCT03126032) and in the ICU (Analysis of Sublingual Glycocalyx Damage at ICU Admission to Predict Risk of Death—the ASGARD Study, Clinicaltrials.gov Identifier: NCT03847493). In these studies, serial measurements can possibly reveal differential changes in individual components of the MVHS in response to the initial therapy.

### Limitations

We acknowledge some limitations of our study. First, it is a single-center study with a limited sample size. Therefore, the findings cannot be directly generalized for all sepsis patients. However, the interdependencies between our novel variables and their associations with clinical variables remained significant after multiple adjustments and additional sensitivity analyses. Second, this study was neither designed nor powered to test the performance of novel variables for outcome prediction. However, our findings add interesting new aspects to the lively field of intravital microscopy research. Further longitudinal studies are needed to evaluate the MVHS for outcome prediction. Third, since all calculations are ultimately based on the flow properties of RBCs, we can only analyze microvessels in which a minimal number of RBCs are present and the predefined quality criteria are met. Vessels without RBCs or invalid vascular segments are therefore not detected by the software. That could have an impact not only on the density calculations, but also on the estimation of the PBR, as severely affected capillaries might not be accessible to RBCs anymore. However, past studies have shown, that the average PBR (4–25 µm) of healthy and septic subjects measured in vivo correlates excellently with the decrease in glycocalyx thickness of endothelial cells after incubation with the concordant serum samples, further supporting the accuracy of PBR values. Fourth, technical limitations, as, e.g., pressure artifacts or low spatial resolution might have affected the results. However, all measurements were performed by only one very experienced investigator specially trained to avoid pressure artifacts. Moreover, the camera has a sufficiently high resolution.

## Conclusion and outlook

We used a highly differentiated analysis to quantify RBC dynamics in sublingual microvessels and found that taking the interdependence between capillary blood volume, capillary recruitment and PBR into account, is key to better understand microvascular dysfunction in sepsis. The MVHS_dynamic_ reflects an impairment of very small capillaries and has a broad bandwidth to detect microvascular dysfunction in critically ill patients. Future clinical studies should evaluate the prognostic value of MVHS in sepsis.

## Supplementary Information

**Additional file 1: Fig. S1**. Screenshots of randomly selected videos of one healthy (A-B) and four sepsis (C-J) individuals. Left: Screenshots without showing automatic vessel detection. Right: Vessels with diameter between 4 and 25 µm of the images on the left column are automatically highlighted and subjected to an automatic quality check. Invalid vascular segments are marked yellow and are automatically discarded, while all valid vascular segments (green lines) are further analyzed (see Figure 1).

**Additional file 2: Video 1**. Measurement of a healthy individual with and without automatic vessel detection and segments’ analysis.

**Additional file 3: Video 2**. Measurement of a sepsis patient with and without automatic vessel detection and segments’ analysis.

**Additional file 4: Fig. S2**. Correlation between manually and automatically measurement of V_RBC_. Average manually derived longitudinal RBC movement per vessel was plotted against automatically measured V_RBC_ of the same specific vessel (Spearman correlation). For manual validation of V_RBC_, 15 vessels (diameter 5 to 17 µm) from 3 randomly selected movies were analyzed manually. Therefore, longitudinal movement of individual RBCs in that vessels were tracked and measured as pixels per frame through subsequent vascular segments. Measured pixels per frame were transformed to µm/sec using camera frame-rate and pixel size.

**Additional file 5: Fig. S3**. Derivation of capillary recruitment. The capillary recruitment is calculated by the slope of the relationship between V_RBC_ (D ≤ 7 µm) and V_RBC_ (D ≥ 10 µm). Two examples: In case capillary blood volume doubles when large vessel RBC velocity increases 2-fold, the slope (V_RBC_ (D ≤ 7 µm), V_RBC_ (D ≥ 10 µm)) will be 0 and CR = 1 – slope 0 = 1 = 100%. In the absence of changes in capillary blood volume when V_RBC_ (D ≥ 10 µm) increases 2-fold, capillary RBC velocities are expected to also change proportionally by 2-fold, and the slope of V_RBC_ (D ≤ 7 µm) vs. V_RBC_ (D ≥ 10 µm) will be 1 and CR = 1 – slope 1 = 0 = 0%.

**Additional file 6: Fig. S4**. Derivation of PBR_dynamic_ from PBR_static_. All dots are shifted to V = 0 µm/sec parallelly to the slopes of each group.

**Additional file 7: Table A1**. Comorbidities, focus of infection and isolated pathogens in the septic cohort. **Table A2**. Correlation coefficient between microvascular and clinical variables shown in Figure 1B, D, F. **Table A3**. Sensitivity analysis in sepsis patients and healthy controls. **Table A4**. Sensitivity analysis in sepsis patients.

**Additional file 8: Fig. S5**. Capillary dropout in sepsis patients. Bar charts showing the percentage of loss of vascular density in sepsis patients compared to healthy controls. Q value (adjusted P value): *q < 0.05, **q < 0.01, ***q < 0.001

**Additional file 9: Fig. S6**. Pooled PBR_static_ values in predefined diameter ranges Boxplots of PBRstatic values of healthy controls and septic patients based on the different microvascular diameter ranges as output by the previous version of the GlyoCheck™ software used in [20].

**Additional file 10: Fig. S7**. Conceptional development of the dynamic version of Microvascular Health score (MVHS_dynamic_)

**Additional file 11: Fig. S8**. Association of MVHS_dynamic_ with numbers of dysfunctional organs in sepsis patients (red) after dichotomizing (median) the group.

## Data Availability

The datasets used and/or analyses during the current study are available from the corresponding author on reasonable request.

## Abbreviations

ASGARD: Analysis of Sublingual Glycocalyx Damage at ICU Admission to Predict Risk of Death
BMI: Body mass index
CBV: Capillary blood volume
CCI score: Charlson Comorbidity Index
CR: Capillary recruitment
CRP: C-reactive protein
D: Diameter
EDGE: Early detection of glycocalyx damage in emergency room patients
FDR: False discovery rate
Hb: Hemoglobin
ICU: Intensive care unit
IL-6: Interleukin-6
IQR: Interquartile range
MAP: Mean arterial pressure
MFI: Microvascular flow index
MVHS: Microvascular health score
PBR: Perfused boundary region
PCT: Procalcitonin
PVD: Perfused vascular density
RBC: Red blood cell
RBCW: Red blood cell width
SDF: Sidestream dark field
SOFA score: Sequential Organ Failure Assessment score
TVD: Total vascular density
UKM: University Hospital Münster
V_RBC_: Red blood cell velocity
WBC: White blood cell
