# Supplementary figures and images for: Identification of novel sublingual parameters to analyze and diagnose microvascular dysfunction in sepsis: the NOSTRADAMUS study
Source: Crit Care. 2021 Mar 19;25:112. doi: 10.1186/s13054-021-03520-w (PMC7980588; doi:10.1186/s13054-021-03520-w)

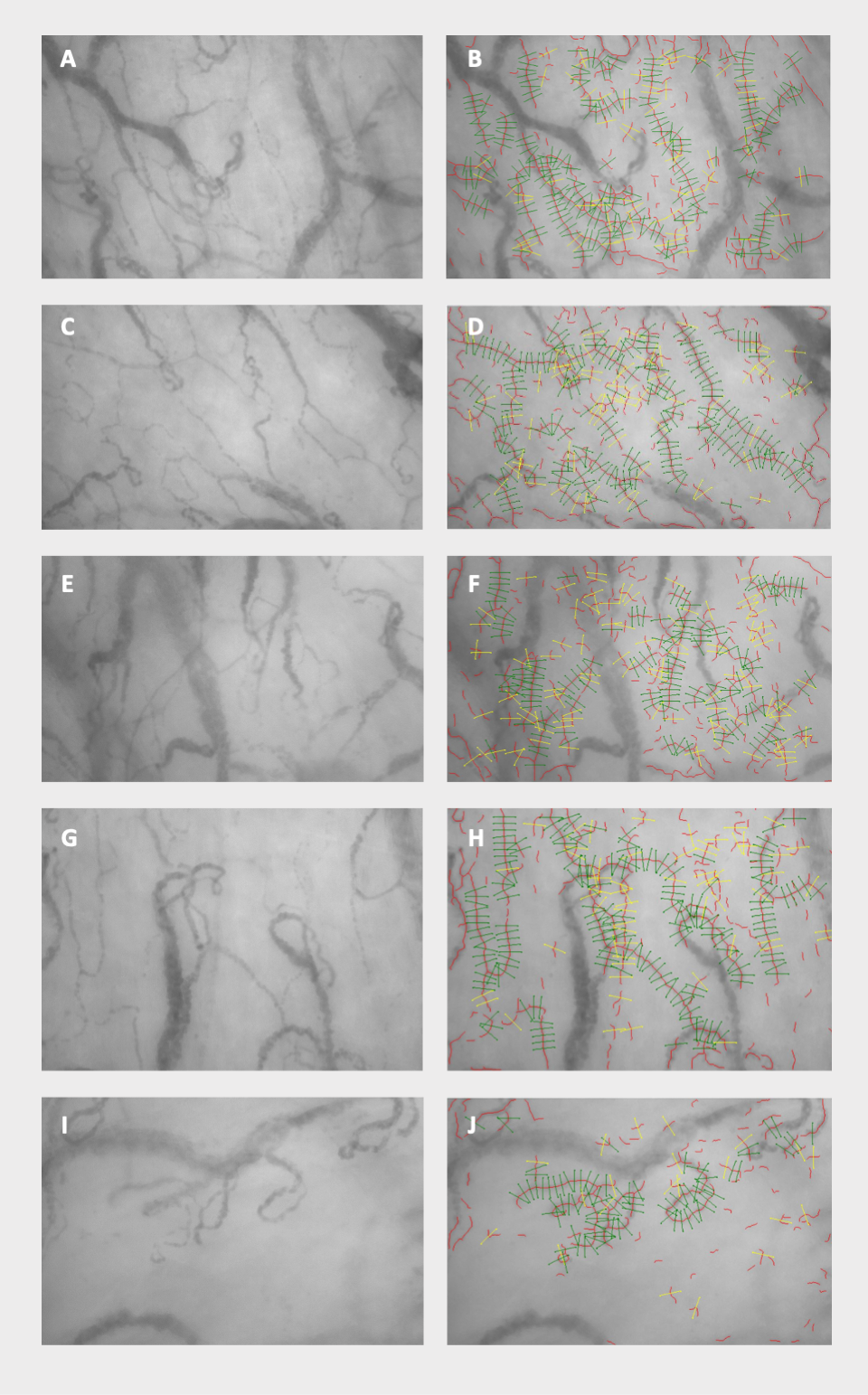

Supplement: Supplementary file 1 — Additional file 1: Fig. S1. Screenshots of randomly selected videos of one healthy (A-B) and four sepsis (C-J) individuals. Left: Screenshots without showing automatic vessel detection. Right: Vessels with diameter between 4 and 25 µm of the images on the left column are automatically highlighted and subjected to an automatic quality check. Invalid vascular segments are marked yellow and are automatically discarded, while all valid vascular segments (green lines) are further analyzed (see Figure 1). [file 13054_2021_3520_MOESM1_ESM.tiff]

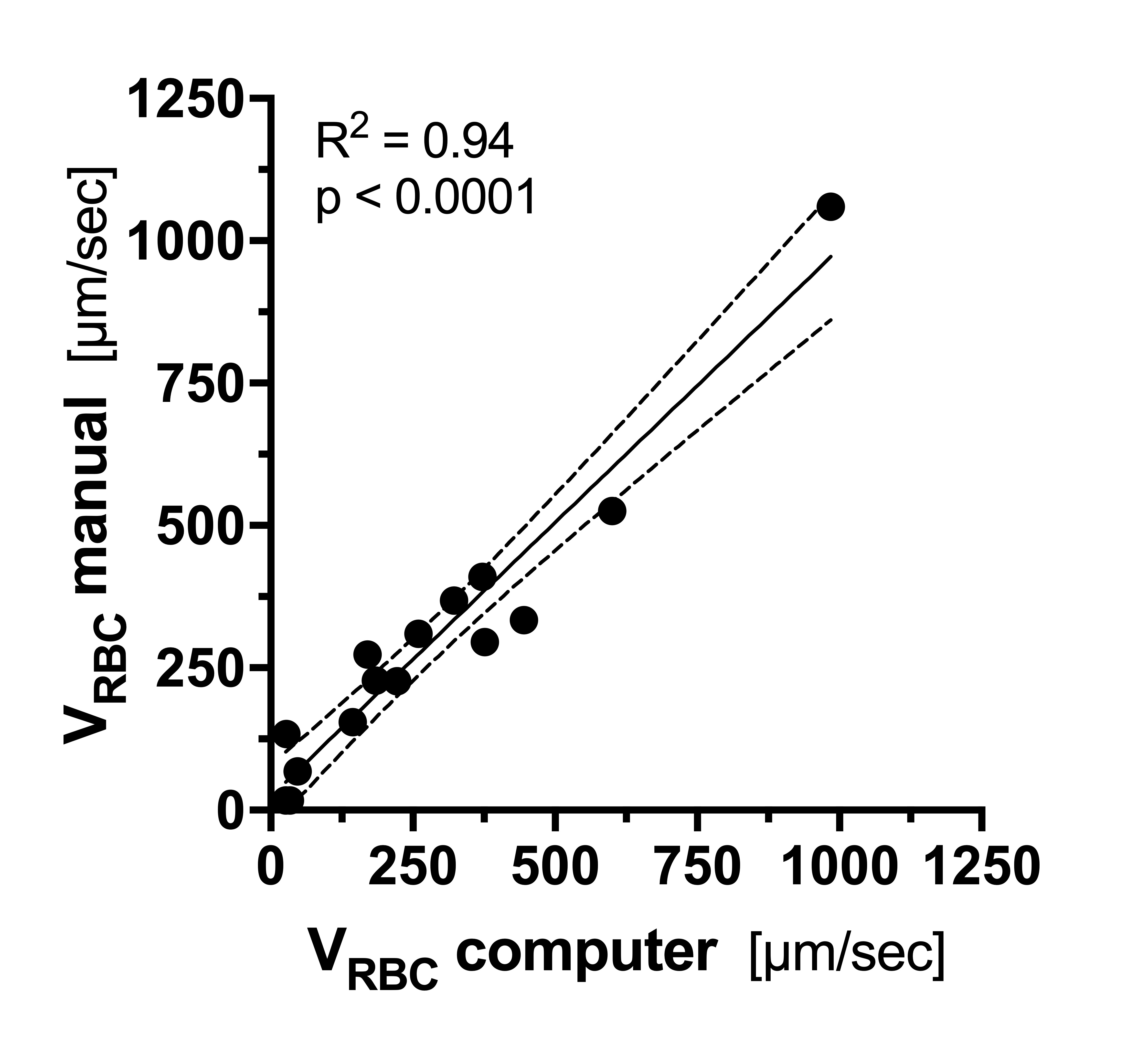

Supplement: Supplementary file 4 — Additional file 4: Fig. S2. Correlation between manually and automatically measurement of VRBC. Average manually derived longitudinal RBC movement per vessel was plotted against automatically measured VRBC of the same specific vessel (Spearman correlation). For manual validation of VRBC, 15 vessels (diameter 5 to 17 µm) from 3 randomly selected movies were analyzed manually. Therefore, longitudinal movement of individual RBCs in that vessels were tracked and measured as pixels per frame through subsequent vascular segments. Measured pixels per frame were transformed to µm/sec using camera frame-rate and pixel size. [file 13054_2021_3520_MOESM4_ESM.jpg]

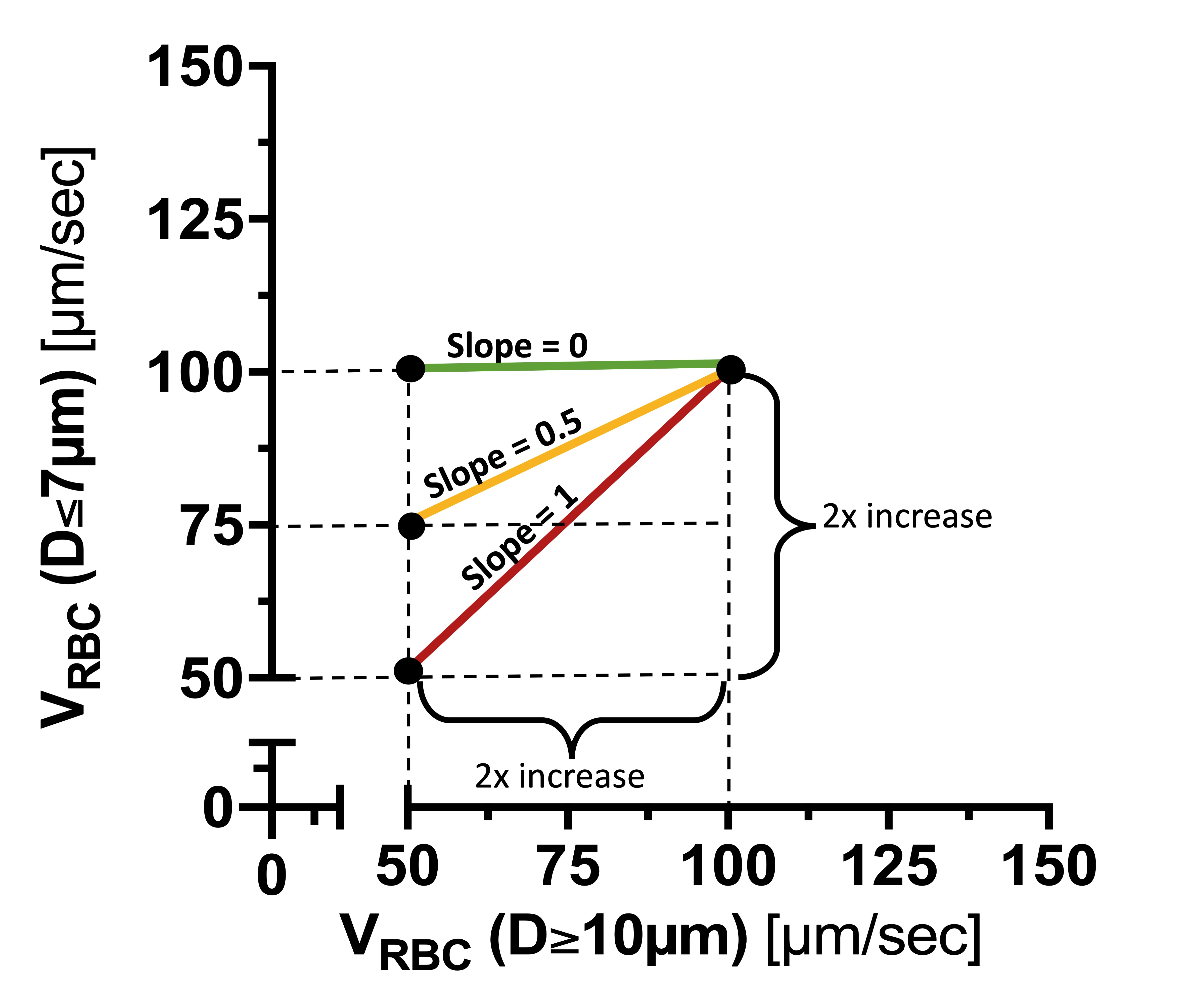

Supplement: Supplementary file 5 — Additional file 5: Fig. S3. Derivation of capillary recruitment. The capillary recruitment is calculated by the slope of the relationship between VRBC (D ≤ 7 µm) and VRBC (D ≥ 10 µm). Two examples: In case capillary blood volume doubles when large vessel RBC velocity increases 2-fold, the slope (VRBC (D ≤ 7 µm), VRBC (D ≥ 10 µm)) will be 0 and CR = 1 – slope 0 = 1 = 100%. In the absence of changes in capillary blood volume when VRBC (D ≥ 10 µm) increases 2-fold, capillary RBC velocities are expected to also change proportionally by 2-fold, and the slope of VRBC (D ≤ 7 µm) vs. VRBC (D ≥ 10 µm) will be 1 and CR = 1 – slope 1 = 0 = 0%. [file 13054_2021_3520_MOESM5_ESM.jpg]

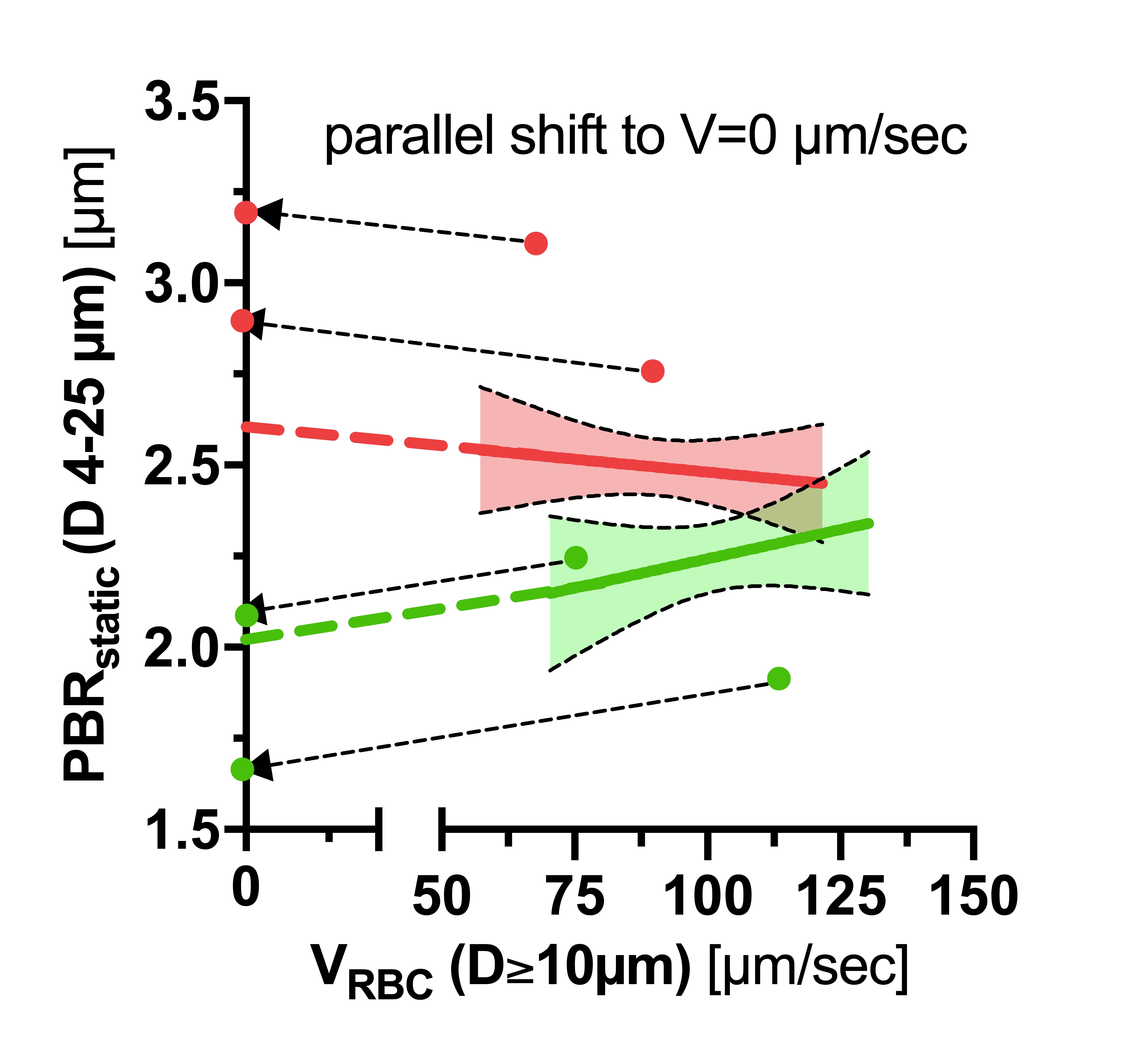

Supplement: Supplementary file 6 — Additional file 6: Fig. S4. Derivation of PBRdynamic from PBRstatic. All dots are shifted to V = 0 µm/sec parallelly to the slopes of each group. [file 13054_2021_3520_MOESM6_ESM.jpg]

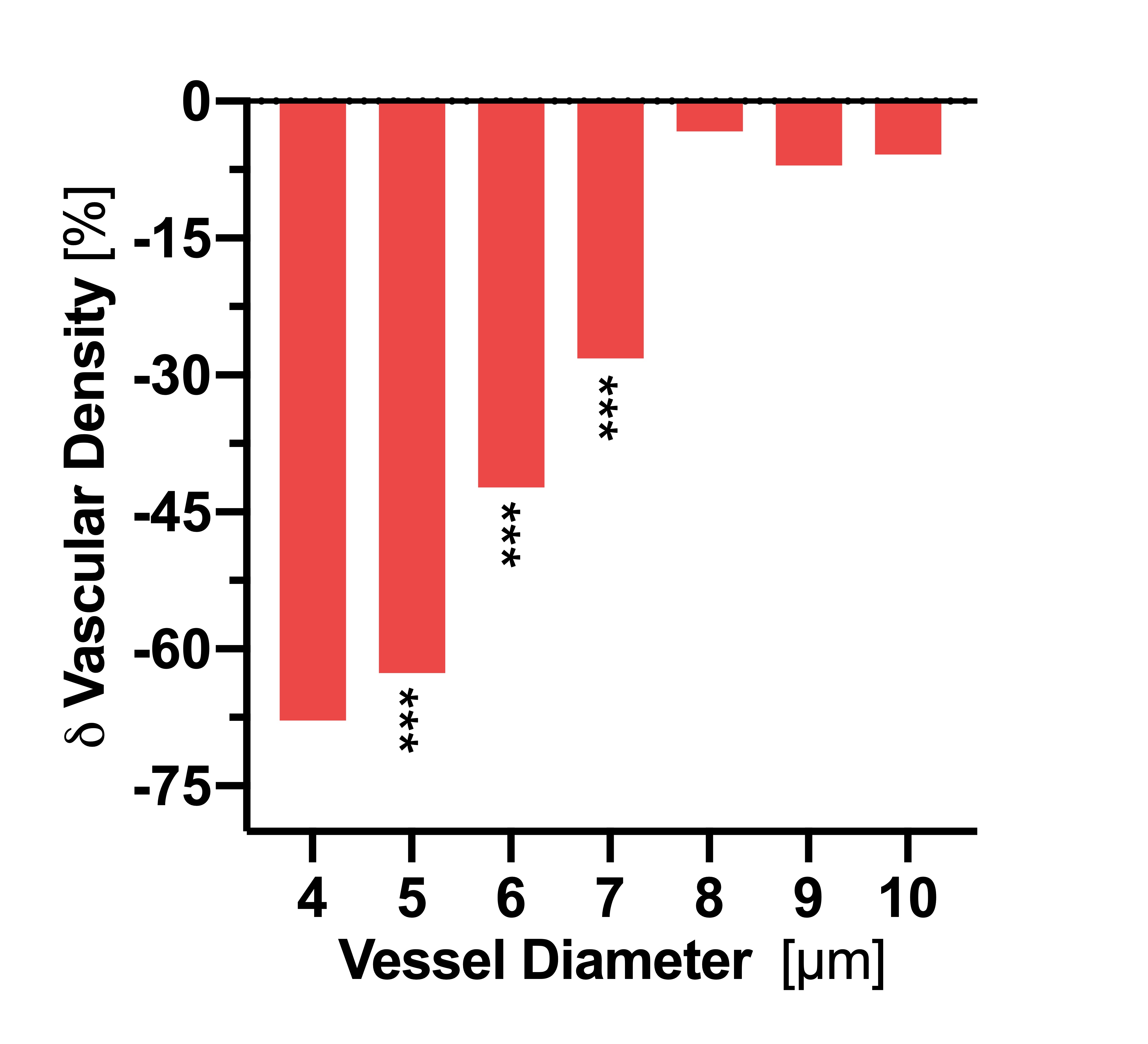

Supplement: Supplementary file 8 — Additional file 8: Fig. S5. Capillary dropout in sepsis patients. Bar charts showing the percentage of loss of vascular density in sepsis patients compared to healthy controls. Q value (adjusted P value): *q < 0.05, **q < 0.01, ***q < 0.001 [file 13054_2021_3520_MOESM8_ESM.jpg]

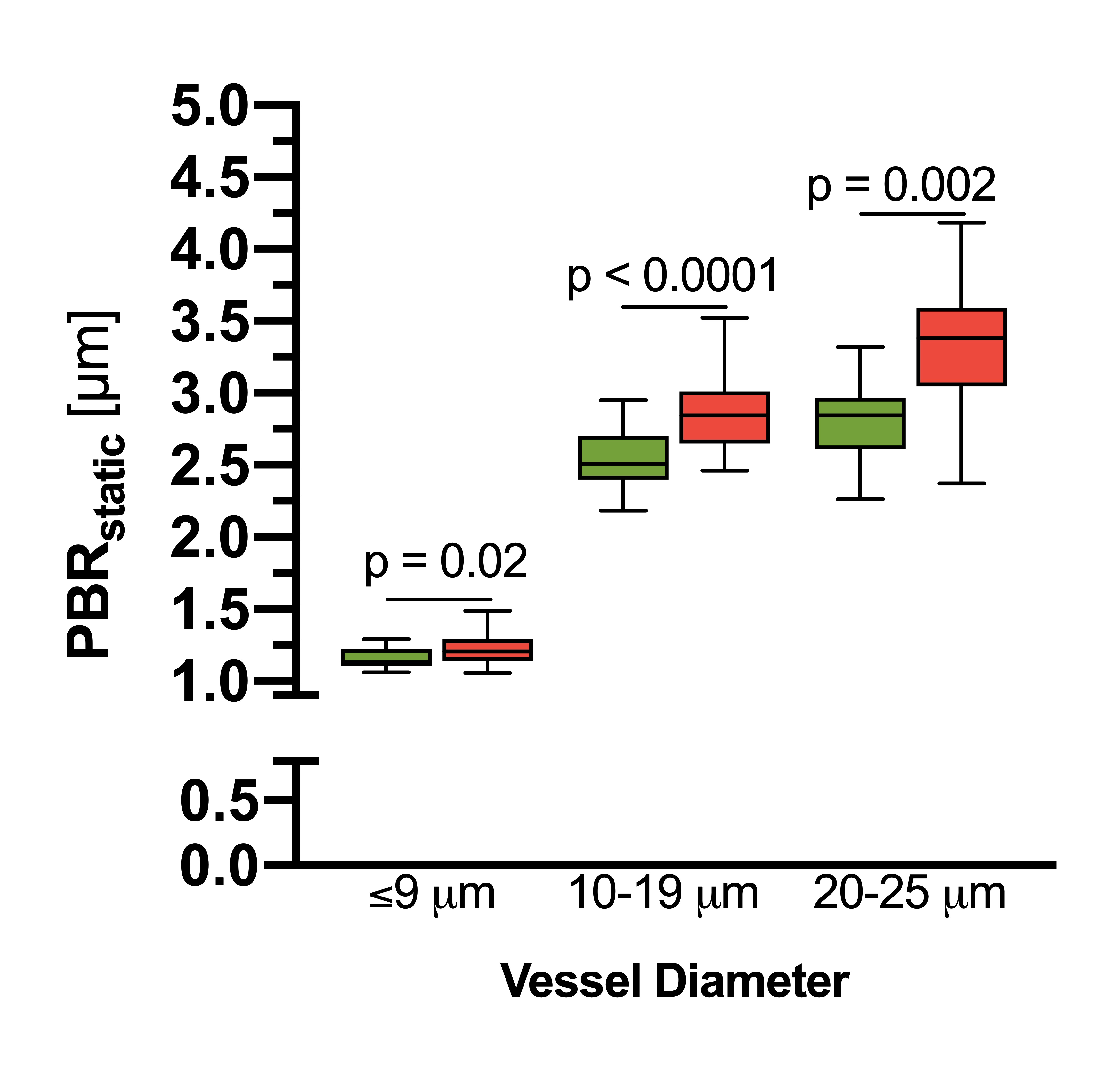

Supplement: Supplementary file 9 — Additional file 9: Fig. S6. Pooled PBRstatic values in predefined diameter ranges Boxplots of PBRstatic values of healthy controls and septic patients based on the different microvascular diameter ranges as output by the previous version of the GlyoCheck™ software used in [20]. [file 13054_2021_3520_MOESM9_ESM.jpg]

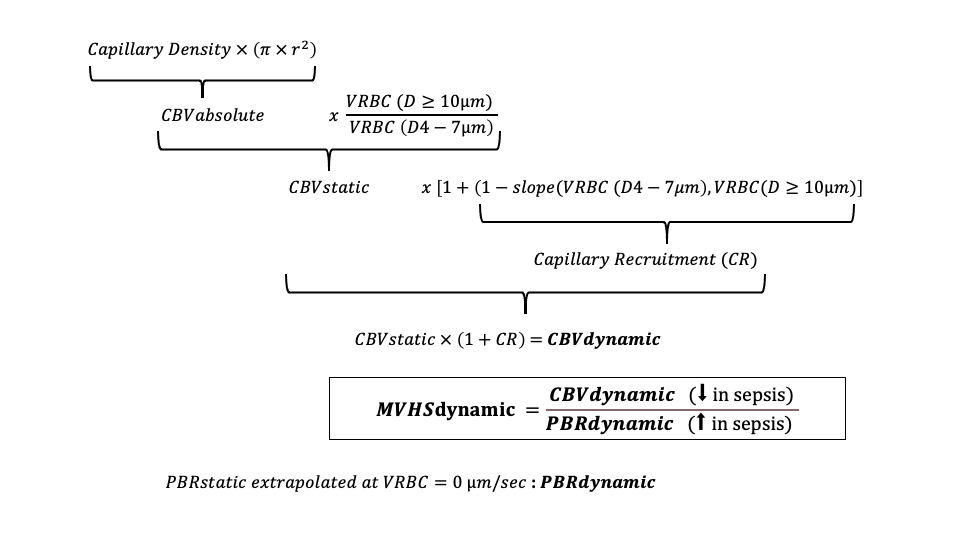

Supplement: Supplementary file 10 — Additional file 10: Fig. S7. Conceptional development of the dynamic version of Microvascular Health score (MVHSdynamic) [file 13054_2021_3520_MOESM10_ESM.tiff]

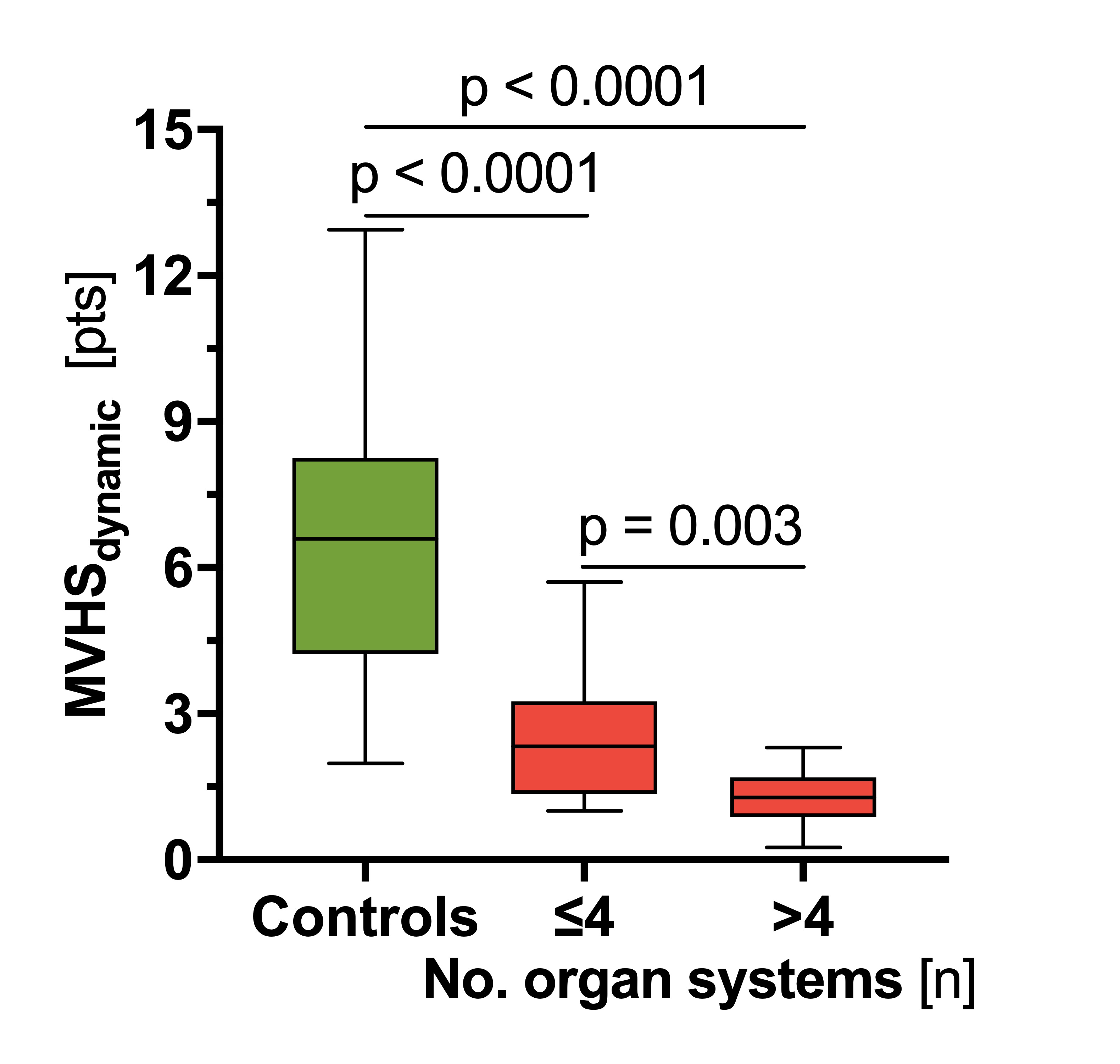

Supplement: Supplementary file 11 — Additional file 11: Fig. S8. Association of MVHSdynamic with numbers of dysfunctional organs in sepsis patients (red) after dichotomizing (median) the group. [file 13054_2021_3520_MOESM11_ESM.jpg]
