# Supplementary material for: Identification of novel sublingual parameters to analyze and diagnose microvascular dysfunction in sepsis: the NOSTRADAMUS study
Source: Crit Care. 2021 Mar 19;25:112. doi: 10.1186/s13054-021-03520-w (PMC7980588; doi:10.1186/s13054-021-03520-w)
Supplement: Supplementary file 7 — Additional file 7: Table A1. Comorbidities, focus of infection and isolated pathogens in the septic cohort. Table A2. Correlation coefficient between microvascular and clinical variables shown in Figure 1B, D, F. Table A3. Sensitivity analysis in sepsis patients and healthy controls. Table A4. Sensitivity analysis in sepsis patients. [file 13054_2021_3520_MOESM7_ESM.docx]

**Identification of** **Novel Sublingual Parameters to Analyze and Diagnose Microvascular Dysfunction in Sepsis – The NOSTRADAMUS Study**

**Running title: The NOSTRADAMUS Study**

Alexandros Rovas^1^, Jan Sackarnd^2^, Jan Rossaint^3^, Stefanie Kampmeier^4^, Hermann Pavenstädt^1^, Hans Vink^5*^, Philipp Kümpers^1*^

^1^Department of Medicine D, Division of General Internal and Emergency Medicine, Nephrology, and Rheumatology, University Hospital Münster, Albert-Schweitzer-Campus 1, 48149 Münster, Germany

^2^Department of Cardiology and Angiology, University Hospital Münster, Albert-Schweitzer-Campus 1, 48149 Münster, Germany

^3^Department of Anesthesiology, Intensive Care and Pain Medicine, University Hospital Münster, Albert-Schweitzer-Campus 1, 48149 Münster, Germany

^4^Institute of Hygiene, University Hospital Münster, Albert-Schweitzer-Campus 1, 48149 Münster, Germany

^5^Department of Physiology, Cardiovascular Research Institute Maastricht, Maastricht University, Maastricht, The Netherlands

*contributed equally and are both considered senior authors

**Additional Tables:**

**Table A1:** Comorbidities, focus of infection and isolated pathogens in the septic cohort.

| **Comorbidities** (n; %) | |
| --- | --- |
| Chronic respiratory disease | 10 (29.4) |
| Congestive heart failure | 15 (44.1) |
| Moderate/severe chronic hepatic disease | 5 (14.7) |
| Dialysis-dependent CKD | 1 (2.9) |
| Malignancy | 6 (17.6) |
| Diabetes Mellitus | 6 (17.6) |
| **Focus of infection** (n; %) | |
| Respiratory tract | 17 (50) |
| Prosthesis | 4 (11.8) |
| Gastrointestinal tract | 3 (8.8) |
| Skin | 2 (5.9) |
| CNS | 2 (5.9) |
| Heart | 1 (2.9) |
| Urinary tract | 1 (2.9) |
| Unknown/Other | 4 (11.8) |
| **Pathogens (genus)** (n) | |
| Klebsiella | 10 |
| Staphylococcus | 8 |
| Enterococcus | 5 |
| Streptococcus | 4 |
| E. Coli | 9 |
| Pseudomonas | 2 |
| Others | 13 |

**Table A2:** Correlation coefficient between microvascular and clinical variables shown in Figure 1B, D, F in vessels with diameter between 4 and 25 µm

|  | **IL6** | **SOFA Score** | **Lactate** | **PCT** |
| --- | --- | --- | --- | --- |
| **D4** | -0.231 | -0.359^**^ | -0.193 | -0.207 |
| **D5** | -0.441^**^ | -0.494^**^ | -0.343^*^ | -0.396^**^ |
| **D6** | -0.450^**^ | -0.507^**^ | -0.384^**^ | -0.386^**^ |
| **D7** | -0.309^*^ | -0.415^**^ | -0.327^*^ | -0.270 |
| **D8** | -0.037 | -0.203 | -0.070 | -0.016 |
| **D9** | -0.005 | -0.131 | -0.027 | 0.029 |
| **D10** | 0.094 | -0.098 | 0.156 | 0.111 |
| **D11** | 0.209 | -0.039 | 0.246 | 0.204 |
| **D12** | 0.185 | 0.034 | 0.183 | 0.157 |
| **D13** | 0.270 | 0.029 | 0.213 | 0.210 |
| **D14** | 0.183 | 0.043 | 0.197 | 0.147 |
| **D15** | 0.161 | -0.041 | 0.116 | 0.047 |
| **D16** | 0.202 | -0.054 | 0.236 | 0.073 |
| **D17** | 0.243 | -0.029 | 0.257 | 0.074 |
| **D18** | 0.200 | -0.015 | 0.244 | 0.109 |
| **D19** | 0.054 | -0.188 | 0.046 | -0.072 |
| **D20** | 0.099 | -0.121 | 0.181 | 0.005 |
| **D21** | 0.067 | -0.165 | 0.026 | -0.017 |
| **D22** | 0.066 | -0.133 | 0.154 | -0.042 |
| **D23** | 0.050 | -0.186 | 0.198 | -0.018 |
| **D24** | 0.009 | -0.171 | 0.066 | -0.037 |
| **D25** | -0.004 | -0.219 | 0.134 | 0.020 |

|  | **IL6** | **SOFA Score** | **Lactate** | **PCT** |
| --- | --- | --- | --- | --- |
| **VRBC4** | -0.026 | -0.187 | -0.007 | -0.100 |
| **VRBC5** | -0.111 | -0.161 | -0.152 | -0.130 |
| **VRBC6** | -0.455^**^ | -0.551^**^ | -0.093 | -0.443^**^ |
| **VRBC7** | -0.223 | -0.280^*^ | -0.051 | -0.220 |
| **VRBC8** | -0.219 | -0.428^**^ | -0.096 | -0.279^*^ |
| **VRBC9** | -0.082 | -0.303^*^ | -0.064 | -0.177 |
| **VRBC10** | -0.296^*^ | -0.505^**^ | -0.166 | -0.367^**^ |
| **VRBC11** | -0.257 | -0.465^**^ | 0.019 | -0.412^**^ |
| **VRBC12** | -0.110 | -0.308^*^ | 0.139 | -0.279^*^ |
| **VRBC13** | -0.034 | -0.200 | 0.050 | -0.069 |
| **VRBC14** | -0.102 | -0.343^*^ | 0.116 | -0.221 |
| **VRBC15** | 0.068 | -0.147 | 0.022 | -0.031 |
| **VRBC16** | 0.030 | -0.206 | 0.161 | -0.216 |
| **VRBC17** | 0.031 | -0.220 | 0.101 | -0.133 |
| **VRBC18** | -0.047 | -0.199 | -0.020 | -0.241 |
| **VRBC19** | -0.096 | -0.189 | -0.084 | -0.201 |
| **VRBC20** | -0.080 | -0.269 | -0.045 | -0.250 |
| **VRBC21** | -0.155 | -0.136 | -0.139 | -0.257 |
| **VRBC22** | 0.053 | -0.077 | -0.002 | -0.114 |
| **VRBC23** | -0.146 | -0.295 | -0.155 | -0.305^*^ |
| **VRBC24** | 0.280 | 0.127 | 0.160 | 0.195 |
| **VRBC25** | -0.025 | -0.039 | -0.068 | -0.083 |

|  | **IL6** | **SOFA Score** | **Lactate** | **PCT** |
| --- | --- | --- | --- | --- |
| **PBR4** | -0.003 | -0.099 | 0.293 | -0.016 |
| **PBR5** | 0.188 | 0.290^*^ | 0.159 | 0.277^*^ |
| **PBR6** | 0.433^**^ | 0.264 | 0.424^**^ | 0.391^**^ |
| **PBR7** | 0.307^*^ | 0.167 | 0.335^*^ | 0.215 |
| **PBR8** | 0.204 | 0.071 | 0.145 | 0.098 |
| **PBR9** | 0.188 | 0.128 | 0.201 | 0.203 |
| **PBR10** | 0.268 | 0.198 | 0.248 | 0.138 |
| **PBR11** | 0.291 | 0.136 | 0.148 | 0.191 |
| **PBR12** | 0.572^**^ | 0.404^**^ | 0.373^**^ | 0.398^**^ |
| **PBR13** | 0.213 | 0.245 | 0.079 | 0.240 |
| **PBR14** | 0.241 | 0.184 | 0.095 | 0.206 |
| **PBR15** | 0.346^*^ | 0.377^**^ | 0.227 | 0.455^**^ |
| **PBR16** | 0.514^**^ | 0.468^**^ | 0.226 | 0.503^**^ |
| **PBR17** | 0.484^**^ | 0.400^**^ | 0.488^**^ | 0.443^**^ |
| **PBR18** | 0.416^**^ | 0.192 | 0.361^**^ | 0.266 |
| **PBR19** | 0.323^*^ | 0.156 | 0.275 | 0.231 |
| **PBR20** | 0.455^**^ | 0.344^*^ | 0.402^**^ | 0.409^**^ |
| **PBR21** | 0.491^**^ | 0.248 | 0.392^**^ | 0.321^*^ |
| **PBR22** | 0.064 | 0.096 | 0.100 | 0.030 |
| **PBR23** | 0.328^*^ | 0.119 | 0.369^*^ | 0.165 |
| **PBR24** | 0.272 | 0.256 | 0.270 | 0.227 |
| **PBR25** | -0.011 | 0.093 | -0.206 | -0.086 |

D = density, IL6 = interleukin 6, PBR = perfused boundary region, PCT = procalcitonin, VRBC = red blood cell velocity, SOFA score = sequential organ failure assessment score. * p < 0.05, ** p < 0.01

**Table A3:** Sensitivity analysis in sepsis patients and healthy controls.

| **Independent Variable** | **MVHS_static_** | | **MVHS_dynamic_** | |
| --- | --- | --- | --- | --- |
|  | Standardized  Coefficient β | *p*  value | Standardized  Coefficient β | *p*  value |
| *SOFA Score unadjusted* | -0.55 | <0.0001 | -0.74 | <0.0001 |
| *SOFA Score adjusted for:*  - Age, sex, BMI, CCI | -0.63 | 0.002 | -0.65 | <0.0001 |
| - Lactate, IL-6, PCT, Hb | -0.34 | 0.10 | -0.34 | 0.03 |
| *Number of dysfunctional organs unadjusted* | -0.56 | <0.0001 | -0.76 | <0.0001 |
| *Number of dysfunctional organs adjusted for:*  - Age, sex, BMI, CCI | -0.70 | 0.001 | -0.73 | <0.0001 |
| - Lactate, IL-6, PCT, Hb | -0.37 | 0.10 | -0.38 | 0.02 |

Linear regression was calculated with MVHS (static or dynamic) used as the dependent variable.

BMI = body mass index, CCI score = Charlson Comorbidity Index, Hb = haemoglobin, IL-6, = interleukin 6, MVHS = microvascular health score, SOFA score = sequential organ failure assessment score

**Table A4:** Sensitivity analysis in sepsis patients.

| **Independent Variable** | **MVHS_static_** | | **MVHS_dynamic_** | |
| --- | --- | --- | --- | --- |
|  | Standardized  Coefficient β | *p*  value | Standardized  Coefficient β | *p*  value |
| *SOFA Score unadjusted* | -0.49 | 0.004 | -0.49 | 0.004 |
| *SOFA Score adjusted for:*  - Age, sex, BMI, CCI | -0.46 | 0.02 | -0.45 | 0.02 |
| - Lactate, IL-6, PCT, Hb | -0.48 | 0.02 | -0.48 | 0.02 |
| *Number of dysfunctional organs unadjusted* | -0.53 | 0.001 | -0.53 | 0.001 |
| *Number of dysfunctional organs adjusted for:*  - Age, sex, BMI, CCI | -0.48 | 0.01 | -0.48 | 0.01 |
| - Lactate, IL-6, PCT, Hb | -0.56 | 0.005 | -0.56 | 0.005 |

Linear regression was calculated with MVHS (static or dynamic) used as the dependent variable.

BMI = body mass index, CCI score = Charlson Comorbidity Index, Hb = haemoglobin, MAP = mean arterial pressure, MVHS = microvascular health score, SOFA score = sequential organ failure assessment score
